# Non-canonical translation in RNA viruses

**DOI:** 10.1099/vir.0.042499-0

**Published:** 2012-07

**Authors:** Andrew E. Firth, Ian Brierley

**Affiliations:** Division of Virology, Department of Pathology, University of Cambridge, Tennis Court Road, Cambridge CB2 1QP, UK

## Abstract

Viral protein synthesis is completely dependent upon the translational machinery of the host cell. However, many RNA virus transcripts have marked structural differences from cellular mRNAs that preclude canonical translation initiation, such as the absence of a 5′ cap structure or the presence of highly structured 5′UTRs containing replication and/or packaging signals. Furthermore, whilst the great majority of cellular mRNAs are apparently monocistronic, RNA viruses must often express multiple proteins from their mRNAs. In addition, RNA viruses have very compact genomes and are under intense selective pressure to optimize usage of the available sequence space. Together, these features have driven the evolution of a plethora of non-canonical translational mechanisms in RNA viruses that help them to meet these challenges. Here, we review the mechanisms utilized by RNA viruses of eukaryotes, focusing on internal ribosome entry, leaky scanning, non-AUG initiation, ribosome shunting, reinitiation, ribosomal frameshifting and stop-codon readthrough. The review will highlight recently discovered examples of unusual translational strategies, besides revisiting some classical cases.

## Introduction

**Fig. 1.**
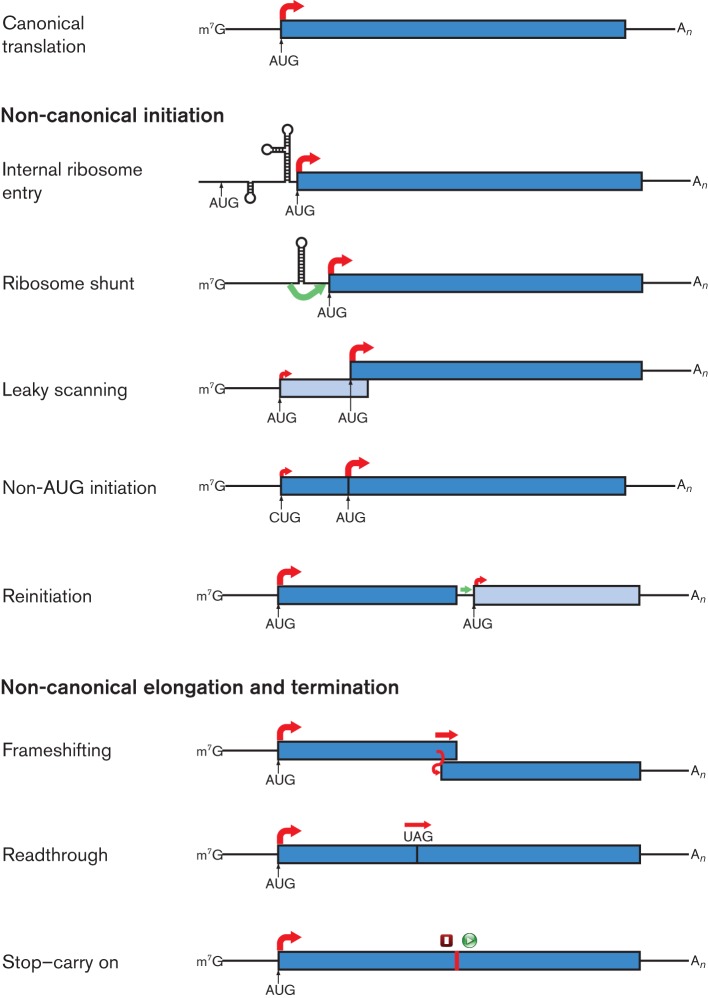
Examples of non-canonical translational mechanisms utilized by RNA viruses. Canonical eukaryotic mRNA translation is shown in the top panel. Red arrows indicate initiation of protein synthesis (at the start of an ORF) or continuation of translation by 80S ribosomes, with thicker arrows indicating the predominant path taken by ribosomes (not to scale). Green arrows indicate the probable movement of 40S subunits in a non-canonical manner. Where two distinct polypeptides are synthesized, the ORFs are shown in different shades of blue; where a recoding event during elongation leads to C-terminal extension of a polypeptide, the two ORFs are shown in the same colour. In the stop–carry on mechanism, both termination and initiation steps are non-canonical, as indicated by the red square and green circle.

No virus encodes its own ribosome. Indeed, it has been proposed that the distinction between cellular life and the virus world could be based simply on whether an entity encodes ribosomes or a capsid (Raoult & Forterre, 2008). Nevertheless, whilst viruses seem to be almost entirely dependent upon their hosts for the provision of components of the translational machinery, they have evolved a profusion of non-canonical mechanisms to allow translation to be customized to their specific needs. Indeed, in RNA viruses in particular, non-canonical translation seems to be more the rule than the exception, with some individual viruses employing several different mechanisms. Here, we review the different types of non-canonical translational mechanisms utilized by viruses of eukaryotes, focusing on RNA viruses, but including also examples from retro-transcribing viruses. The focus is on the different translational strategies that RNA viruses employ for accessing multiple ORFs in mRNAs. Such strategies include internal ribosome entry, leaky scanning, non-AUG initiation, ribosome shunting, reinitiation, ribosomal frameshifting and stop-codon readthrough (summarized in Fig. 1). Although several excellent reviews have been written previously on similar topics, the recent explosion in the pace of sequencing has seen many interesting new examples of non-canonical translation come to light in just the past few years. This review will discuss some of these recently discovered examples, besides revisiting some classical cases.

## Canonical translation in eukaryotes

In order to appreciate alternative translational mechanisms, it is instructive to review first the standard course of events in translation of eukaryotic cellular mRNAs, the vast majority of which bear a 5′ cap structure (m^7^G) and a 3′ poly(A) tail. Translation can be divided into four stages: initiation, elongation, termination and ribosome recycling. The initiation step (reviewed by Jackson *et al.*, 2010) begins upon recognition of the 5′ cap structure by initiation factor (eIF) 4E, which recruits eIF4G, a scaffold protein, which in turn engages eIF4A and eIF4B and also the poly(A)-binding protein (PABP). PABP is actually bound at the poly(A) tail at the 3′ end of the mRNA, and its interaction with eIF4G leads to mRNA circularization (see below). The resulting complex of initiation factors recruits the 43S preinitiation complex, comprising the 40S subunit of the ribosome together with eIF3, eIF1, eIF1A, eIF5 and the ternary complex eIF2–Met–tRNA_i_–GTP. This is thought to be largely via interaction between eIF4G and eIF3. Following recruitment to a 5′-proximal position, the preinitiation complex scans along the mRNA until it encounters the first AUG codon. Scanning is assisted by the helicase eIF4A and its cofactor eIF4B, which unwind RNA secondary structures in the 5′UTR of the mRNA. The initiation factors eIF1 and eIF1A are key to the recognition of the AUG codon and its surrounding nucleotide context. Once an AUG codon is recognized, eIF5 triggers hydrolysis of eIF2-bound GTP, the 40S subunit locks into a closed conformation with Met–tRNA_i_ in the ribosomal P-site, and eIF1, eIF2–GDP and eIF5 are released. Then, eIF5B and GTP catalyse joining of the 60S subunit of the ribosome to form the 80S ribosome and release of eIF5B and eIF1A. Translation proceeds to the elongation stage, and eIF3 and its associated eIF4G are thought to be released shortly thereafter.

During elongation, consecutive triplet codons are recognized in the ribosomal A-site by cognate aminoacylated tRNAs delivered to the A-site by eukaryotic elongation factor eEF1A. As each codon is recognized in the A-site, a peptide bond is formed, transferring the nascent polypeptide chain from the P-site tRNA to the A-site tRNA. Translocation, catalysed by eEF2, passes the P-site deacylated tRNA to the E-site (where it is released from the ribosome) and the A-site peptidyl–tRNA to the P-site, thus also moving the mRNA through the ribosome and opening the A-site for the next round of elongation. Elongation continues until a termination codon (UAG, UGA or UAA in the standard genetic code) enters the A-site. These codons are recognized by eukaryotic release factor eRF1, which, together with eRF3 and GTP, mediates termination of translation and release of the newly synthesized protein via hydrolysis of the P-site peptidyl–tRNA (reviewed by Jackson *et al.*, 2012). Post-termination ribosomes are separated into component 40S and 60S subunits by ATP and ABCE1 [a member of the ATP-binding cassette (ABC) family of proteins]. Release of the P-site deacylated tRNA and of the 40S subunit from the mRNA is mediated by eIF3, eIF1 and eIF1A, which are then thought to remain associated with the 40S subunit. The mRNA circularization described earlier is believed to enhance translation, possibly by aiding ribosome recycling and/or by tethering initiation factors to the message (Wells *et al.*, 1998; Jackson *et al.*, 2010).

## Why is non-canonical translation so abundant in viruses?

One of the major challenges facing RNA viruses is the 5′-end dependence of canonical eukaryotic translation initiation, which generally permits the synthesis of only a single protein from a given mRNA. RNA viruses must generally express multiple structural and enzymic proteins to complete their replicative cycle, and they have evolved a variety of strategies to meet this requirement. Some are compatible with 5′-end-dependent translation; for example, the production of functionally monocistronic subgenomic RNAs (sgRNAs; e.g. coronaviruses and closteroviruses) or the use of segmented genomes where most segments are monocistronic (e.g. reoviruses and orthomyxoviruses). Another common strategy is to encode long polyproteins that are subsequently processed by virus-encoded or host proteases to generate the viral proteome (e.g. picornaviruses and flaviviruses). However, the use of these mechanisms has consequences. Viruses with segmented genomes have to ensure the correct packaging of the different segments, or must be able to tolerate the reduction in specific infectivity if segments are packaged randomly or individually into separate virions. Polyprotein expression can be considered to be an inefficient way of exploiting the available resources of the host cell as the mature virus proteins are produced in equal amounts, even though the enzymic proteins are often required in much smaller quantities than the structural proteins. Moreover, while some viral proteins may be expressed from sgRNAs, in probably all positive-strand RNA viruses the components of the replication complex must still be translated from the genomic RNA. Non-canonical translational mechanisms provide a number of alternative ways to express multiple proteins from a single mRNA.

RNA viruses also have very compact genomes, with the largest around 30 kb (e.g. some members of the families *Coronaviridae* and *Reoviridae*). Thus there is strong selective pressure to optimize their coding capacity, for example via the utilization of overlapping ORFs. Non-canonical translational strategies such as leaky scanning, ribosomal frameshifting and alternative initiation are essential in facilitating access to such ORFs. Non-canonical translational mechanisms may also help to overcome the challenges imposed by the marked structural differences present in many viral transcripts in comparison with typical cellular mRNAs. For example, packaging and/or replication signals within the 5′UTR of the genomic RNA or RNA segments can inhibit scanning-dependent translation initiation. Ribosome shunting or internal ribosome entry sites (IRESes) can be employed to circumvent such impediments. Non-canonical translational mechanisms may also play roles in regulating the expression level and/or timing of expression of various proteins.

It should be noted that many RNA viruses lack the machinery to add a 5′ cap and/or poly(A) tail to their transcripts and have evolved alternative mechanisms for ribosome recruitment and/or mRNA circularization. Similarly, several viruses have evolved proteins and or RNA structural elements that further enhance translation of the viral mRNAs. Due to space limitations, such mechanisms will mostly not be discussed here. Many excellent reviews covering these topics, besides some cautionary critiques, are available (Kozak, 2004; Dreher & Miller, 2006; Kneller *et al.*, 2006; Edgil & Harris, 2006; Miller & White, 2006; Kozak, 2007; Miller *et al.*, 2007; Thiébeauld *et al.*, 2007; Nicholson & White, 2011; Walsh & Mohr, 2011).

## Non-canonical initiation

### Internal ribosome entry

IRESes are highly organized, complex RNA structures that recruit ribosomes to internal positions on mRNAs (reviewed by Kieft, 2008; Balvay *et al.*, 2009). In viruses, they are often employed as a way to facilitate translation initiation whilst allowing replication elements and/or packaging signals to be accommodated within the 5′UTR. They may also function to allow translation of viral mRNAs to continue, even when host-cell translation is inhibited, for example by viral protease cleavage of initiation factors required for 5′-cap-dependent translation. IRESes can also be used to access internal ORFs that would otherwise be inaccessible. Viral IRESes vary in both the degree of dependence on initiation factors and the precision with which the initiation site is selected. In picornaviruses, where IRESes were first described (Jang *et al.*, 1988; Pelletier & Sonenberg, 1988), two major classes (types I and II) have been identified that are distinct in structure and sequence, but typically require most of the canonical initiation factors for activity, including eIF3, eIF4A and the C-terminal domain of eIF4G, besides the eIF2–Met–tRNA_i_–GTP ternary complex (reviewed by Belsham, 2009; other types of picornavirus IRES – such as those found in Aichi virus and hepatitis A virus – will not be discussed here). In those picornaviruses harbouring type I IRESes (poliovirus and other enteroviruses), the initiator AUG for translation of the viral polyprotein is located some distance downstream of the site of recruitment of the 40S subunit to the IRES, and some form of scanning is required to locate it. In type II IRESes, found in cardioviruses such as Theiler’s murine encephalomyelitis virus and aphthoviruses such as foot-and-mouth disease virus, the initiator AUG is close to the ribosome entry point and little, if any, scanning is required. In contrast, the IRES of hepatitis C hepacivirus (family *Flaviviridae*) requires fewer initiation factors (eIF3, eIF5 and the eIF2–Met–tRNA_i_–GTP ternary complex), recruits 40S subunits directly, and places the initiator AUG into the ribosomal P-site without any requirement for scanning (reviewed by Lukavsky, 2009). Similar IRESes are also found in some pestiviruses and teschoviruses. Yet other types of IRESes have been described in human immunodeficiency virus (HIV)-1, HIV-2 and other retroviruses (reviewed by Chamond *et al.*, 2010), dicistroviruses (see below) and various other viruses. On a related note, under conditions of eIF2 phosphorylation (which results in global inhibition of translation, often in response to virus infection), initiation may proceed via an eIF2-independent route that involves the cellular protein ligatin, provided there exist structures within the mRNA that position the initiation codon directly in the ribosomal P-site (Skabkin *et al.*, 2010). This mechanism has been demonstrated in the sgRNA of Sindbis alphavirus, and can also occur on the hepatitis C virus IRES.

As the type I and type II picornavirus IRESes do not necessarily place the ribosome directly onto a specific initiation codon, they have the potential to direct initiation at more than one site on the mRNA. One example where a second initiation site is utilized functionally occurs in Theiler’s murine encephalomyelitis virus, where a 156-codon ORF overlaps the 5′ end of the polyprotein ORF in the +1 reading frame (Kong & Roos, 1991; van Eyll & Michiels, 2002). The ORF, which encodes the L* protein, is translated from an AUG codon positioned 13 nt 3′ of the polyprotein AUG initiation codon. In neurovirulent strains, the L* AUG codon is replaced with an ACG codon, but some level of translation still occurs (van Eyll & Michiels, 2002). It has been suggested that translation of L* may be facilitated by the IRES placing a proportion of scanning-competent ribosomes 3′ of the polyprotein initiation codon or otherwise promoting leaky scanning beyond the polyprotein AUG, although the exact mechanism(s) are unknown (van Eyll & Michiels, 2002). A similarly positioned but otherwise unrelated overlapping coding sequence (with an upstream ACG initiator) appears likely to be present in turdivirus 3 (family *Picornaviridae*, genus *Paraturdivirus*). On the other hand, in foot-and-mouth disease virus, two in-frame AUG codons are used as alternative initiation sites to produce different isoforms (Lab and Lb) of the leader protease (reviewed by Belsham, 2005).

**Fig. 2.**
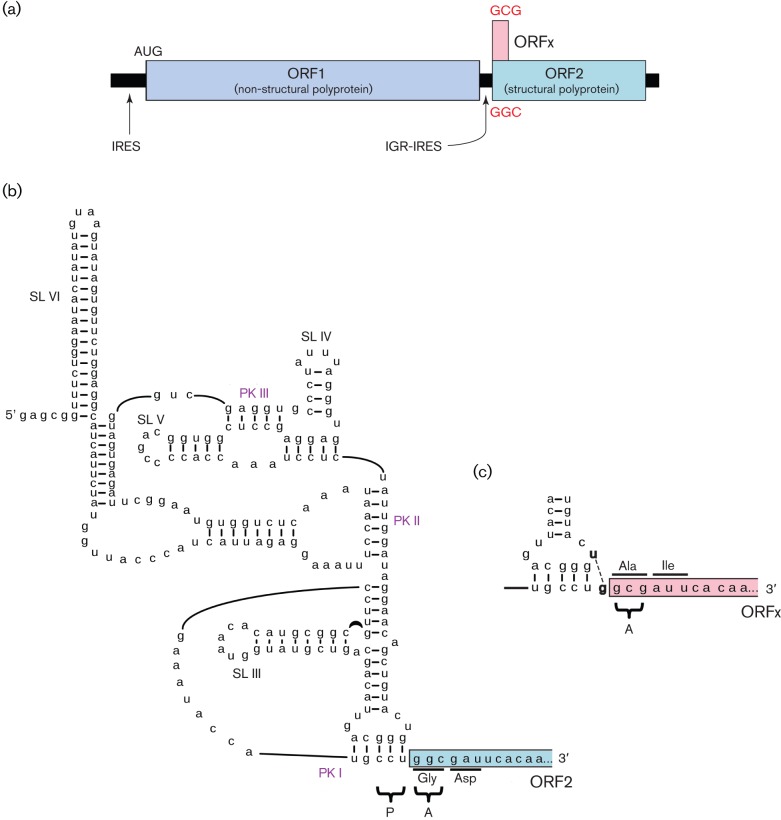
The Israeli acute paralysis dicistrovirus IGR-IRES directs translation of two overlapping ORFs. (a) Genome map. Distinct IRESes direct translation of non-structural and structural polyproteins. The IGR-IRES also directs translation of ORFx, which overlaps ORF2 in the +1 reading frame. (b) Schematic of the IGR-IRES, showing pseudoknots (PK) I, II and III and stem–loops (SL) III, IV, V and VI. PK I occupies the ribosomal P-site and translation of ORF2 initiates at the GGC codon in the ribosomal A-site. (c) The formation of an additional base pair in PK I (U-G; bold) leads instead to initiation at the +1 frame GCG codon and translation of ORFx. Modified from a figure kindly provided by E. Jan (Ren *et al.*, 2012).

Another type of IRES has been described in the genomes of members of the family *Dicistroviridae* – a family of positive-stranded monopartite viruses that infect arthropods. Unusually, dicistroviruses have two non-overlapping coding sequences where translation of each is directed by a distinct IRES. The intergenic region IRES (IGR-IRES) that directs translation of the 3′ ORF encoding structural proteins is very unusual. It is short (typically around 180 nt) in comparison to picornavirus IRESes (typically around 450 nt) and compact, but is folded elegantly by virtue of RNA pseudoknotting into a structure that can partly mimic E- and P-site tRNAs, including the P-site codon : anticodon duplex (Fig. 2). The IGR-IRES binds to ribosome subunits and assembles translationally competent 80S ribosomes, which remarkably can initiate translation on a non-AUG codon in the A-site (in contrast to the P-site in conventional initiation) without any requirement for Met–tRNA_i_ or any of the canonical initiation factors (Wilson *et al.*, 2000; Jang *et al.*, 2009). Precise placement of the ribosome on the mRNA and the absence of initiation factors would seem to preclude any form of leaky scanning in this case. Nonetheless, members of at least one group of dicistroviruses (Israeli acute bee paralysis and related viruses) express an additional protein from a short ORF that overlaps the 5′-proximal region of the structural polyprotein ORF in the +1 reading frame (Ren *et al.*, 2012). Translation of this ORF appears to be directed by an extra base-pairing interaction in the P-site anticodon : codon-mimicking duplex of the IGR-IRES that facilitates a proportion of incoming A-site tRNAs to pair not to the structural polyprotein initiation codon, but instead to the codon offset by +1 nt (Fig. 2; Ren *et al.*, 2012). Besides dicistroviruses, a number of other viruses appear to employ the strategy of expressing two polyproteins from separate IRESes. Canine picodicistrovirus appears to have two picornavirus-like IRESes, with the 3′ ORF encoding the RNA-dependent RNA polymerase (RdRp) and other non-structural proteins (Woo *et al.*, 2012), while several other unclassified dicistronic positive-strand RNA viruses may have a dicistrovirus-like IGR-IRES, although the details have not yet been determined and alternative translation strategies have not been definitively ruled out (Boros *et al.*, 2011).

Hepatitis C virus also has an ORF named core+1 (alternatively F or ARF) that overlaps the 5′-proximal region of the polyprotein ORF (Walewski *et al.*, 2001; reviewed by Vassilaki & Mavromara, 2009). There is little doubt that the core+1 ORF is expressed *in vivo*, at least at some level, as infected patients develop antibodies against core+1 peptides (Walewski *et al.*, 2001). Many different mechanisms have been proposed to account for expression of the core+1 ORF, including ribosomal frameshifting, transcriptional slippage, and independent initiation at either AUG or non-AUG codons. However, which, if any, of these mechanisms are utilized by the virus, and whether the products provide any functional benefit for the virus, remains unclear despite extensive research.

### Leaky scanning

In the scanning model of initiation, 40S ribosomal subunits bind close to the 5′ cap and scan linearly until they encounter the first AUG codon (Kozak, 2002). However, in some circumstances, a significant proportion of scanning ribosomes fail to initiate at the first AUG codon but, instead, continue scanning until they reach an alternative initiation codon further downstream (reviewed by Kozak, 2002). This process is termed leaky scanning and may allow the expression of multiple C-terminally coincident isoforms of a single protein (in-frame alternative initiation sites), distinct proteins encoded by different overlapping ORFs (alternative initiation sites in different reading frames) or even distinct proteins encoded by non-overlapping sequential ORFs. The distance scanned can be considerable – for example, in rice tungro bacilliform tungrovirus, ribosomes scan through a region of around 895 nt to translate the third of three consecutive ORFs (Fütterer *et al.*, 1997). Several other cases have been identified where three or even four distinct proteins are translated from a single transcript, often involving initiation at non-AUG codons (see below), besides AUG codons with poor context (Turina *et al.*, 2000; Castaño *et al.*, 2009). Leaky scanning is perhaps the mechanism most commonly used by RNA viruses to translate functionally multicistronic messages (Table 1). The efficiency of initiation at a potential initiation codon is modulated by its context, i.e. the identity of the nucleotides immediately preceding and immediately following the initiation codon. In mammals, the optimal context includes a G at +4 and a purine at −3 (the initiation codon itself corresponds to nucleotides +1 to +3), where the presence of an A at −3 is the strongest single indicator of efficient initiation (Kozak, 1986). Contexts with an A at −3, or a G at −3 and a G at +4, may be regarded as ‘strong’. Although the optimal context appears to vary between taxa, preference for an A (or G) at the −3 position is widespread in many animals, plants and fungi (Nakagawa *et al.*, 2008). When the context of the first AUG on the message is suboptimal, there is potential for efficient leaky scanning.

**Table 1.** Examples of known and suspected cases of leaky scanning See also Table 2 for cases that involve non-AUG initiation. Full details of references in Tables 1–5 are available from the authors on request.

Taxon	Products	References
*Umbravirus*	ORF3/ORF4	Taliansky & Robinson (2003)
*Luteovirus*	CP/MP	Dinesh-Kumar & Miller (1993)
*Polerovirus*	CP/MP	Tacke *et al.* (1990)
*Polerovirus*	P0/P1	Mayo *et al.* (1989)
*Sobemovirus*	P1/P2a	Sivakumaran & Hacker (1998)
*Tombusvirus*	MP/p20	Johnston & Rochon (1996)
*Aureusvirus*	MP/p14	Rubino & Russo (1997)
*Pelarspovirus*	MP1/MP2/CP	Castaño *et al.* (2009)
*Panicovirus*	MP1/MP2/CP/p15	Turina *et al.* (2000)
*Machlomovirus*	MP1/MP2/CP	Scheets (2000)
*Machlomovirus*	p32/replicase	Nutter *et al.* (1989)
*Tymovirus*	p69/replicase	Weiland & Dreher (1989)
*Potexvirus*	TGB2/TGB3	Verchot *et al.* (1998)
*Hordeivirus* RNA2	TGB2/TGB3	Zhou & Jackson (1996)
*Pecluvirus* RNA2	CP/p39	Herzog *et al.* (1995)
*Arterivirus*	ORF5a/GP5	Firth *et al.* (2011)
*Betacoronavirus* (some species)	N/I	Senanayake *et al.* (1992)
*Hepevirus*	ORF3/CP	Graff *et al.* (2006)
*Caliciviridae* – murine norovirus, neboviruses, some sapoviruses	VP1/ORF4	Simmonds *et al.* (2008); McFadden *et al.* (2011)
*Omegatetravirus* RNA2	p17/CP	Hanzlik *et al.* (1995)
Mammalian orthoreovirus, segment S1	σ1/σ1s	Ernst & Shatkin (1985)
Avian orthoreovirus and Nelson Bay orthoreovirus, segment S1	p10/p17	Racine *et al.* (2007)
Rotavirus A, segment 11	NSP5/NSP6	Mattion *et al.* (1991)
*Respirovirus*	P/C	Giorgi *et al.* (1983)
*Morbillivirus*	P/C	Bellini *et al.* (1985)
*Henipavirus*	P/C	Lo *et al.* (2009)
*Orthobunyavirus* (some species)	N/NSs	Fuller *et al.* (1983)
*Hantavirus* (some species)	N/NSs	Vera-Otarola *et al.* (2012)
*Orthomyxoviridae* – influenza virus B	NB/NA	Williams & Lamb (1989)
*Orthomyxoviridae* – influenza virus A	PB1/PB1-F2/N40	Chen *et al.* (2001); Wise *et al.* (2009)
*Isavirus*, segment 8	P6/P7	Rimstad & Mjaaland (2002)
*Tungrovirus*	ORF I/ORF II/ORF III	Fütterer *et al.* (1997)
*Badnavirus*	ORF I/ORF II/ORF III	Pooggin *et al.* (1999)

One of the earliest examples described is found in segment S1 of mammalian orthoreovirus (Ernst & Shatkin, 1985). Here, the coding sequence for a 14 kDa non-structural protein, σ1s, lies entirely within the coding sequence for the 49 kDa attachment protein, σ1. The σ1 AUG initiation codon has a suboptimal context (cggAUGg) and the σ1s AUG codon is 58 nt 3′ in the +1 reading frame. Another early example of leaky scanning occurs in the small segment of the orthobunyaviruses. Here, the NSs protein is translated from an ORF of around 100 codons that overlaps the 5′-terminal region of the N (nucleocapsid) protein ORF (Fuller *et al.*, 1983). A similar N/NSs arrangement is also present in hantaviruses (Vera-Otarola *et al.*, 2012). Such short, overlapping ORFs, often evolved relatively late, have a more limited phylogenetic distribution than the ancestral ORFs that they overlap, and the encoded proteins tend to have ancillary functions (Rancurel *et al.*, 2009).

Besides suboptimal context surrounding the first AUG codon on a message, leaky scanning may also be promoted by a number of other mechanisms. If an AUG codon is very close to the 5′ end of the transcript, then it is often not recognized efficiently, with the efficiency diminishing as the 5′UTR length decreases below 30 nt, and particularly below 12 nt (Sedman *et al.*, 1990; Kozak, 1991). In murine norovirus (family *Caliciviridae*), translation of an ORF overlapping the capsid coding sequence appears to initiate at the third AUG codon on the sgRNA (underlined in gugaAUGaggAUGagugAUGg; McFadden *et al.*, 2011) despite the presence of two upstream AUG codons, the second of which is in a good context with an A at −3. It seems likely that, in this case, the shortness of the 5′ leader (4 or 10 nt) promotes leaky scanning past the first two AUG codons (which, nonetheless, must also be utilized efficiently for expressing the capsid protein). It should be noted that calicivirus RNAs lack a 5′ cap and instead possess a viral protein (VPg) linked covalently to the 5′ end. The VPg interacts with eIF4E and other initiation factors but, in murine norovirus, the role of these interactions in translation remains uncertain (Daughenbaugh *et al.*, 2006; Chaudhry *et al.*, 2006). It is possible that the presence of VPg facilitates 40S binding on a short leader.

The close proximity of a downstream AUG codon to a preceding AUG codon (e.g. within approx. 10 nt) can also increase the efficiency of leaky scanning. This has been demonstrated in both tymoviruses and in segment 6 of influenzavirus B (4 nt separation; Williams & Lamb, 1989; Matsuda & Dreher, 2006), and may also be relevant to murine norovirus (see above). The data suggest that scanning may involve alternating forward thrusts and backwards relaxations so that downstream AUG codons can sometimes capture a proportion of scanning ribosomes that might otherwise scan back to a slightly upstream AUG codon. [Conversely, initiation at a downstream AUG codon may stimulate initiation at an upstream AUG codon via a mechanism which is thought to depend on a scanning 40S subunit stacking up behind a ribosome initiating at the downstream AUG codon in an appropriate position for initiation at the upstream AUG codon (Dinesh-Kumar & Miller, 1993).]

Leaky scanning can also be promoted by short upstream ORFs. Ribosomes that translate a short ORF have the capacity to resume scanning and reinitiate on a downstream ORF, but it can take time for such ribosomes to reacquire the relevant initiation factors, and some intervening AUG codons may be efficiently bypassed (see also section entitled Reinitiation). Indeed, there are a number of cases of leaky scanning – including the PB1-F2 protein of influenza virus A – where there are one or more intervening AUG codons between the initiation codons of the ORFs that encode functional products (Chen *et al.*, 2001; Wise *et al.*, 2009; Racine & Duncan, 2010). Such AUG codons would be expected to ‘soak up’ a proportion of scanning ribosomes – depending on the strength of their contexts – but, provided the ORFs are short, these ribosomes may still be able to reinitiate on the major downstream ORF(s). Thus, some cases of leaky scanning probably also include an element of reinitiation and possibly also a degree of shunting or non-linear scanning (Racine & Duncan, 2010; see section on ribosome shunting). RNA structure in the scanned region, besides the location, length and amino acid composition of short intervening ORFs, may all influence the proportion of ribosomes that eventually reach the major downstream ORF(s).

### Non-AUG initiation

Eukaryotic protein synthesis begins almost exclusively (but see discussion of the dicistrovirus IGR-IRES above, and see also Skabkin *et al.*, 2010) with methionine, brought to the ribosome by Met–tRNA_i_, a tRNA that differs from the standard (elongation) Met–tRNA. However, initiation does not necessarily have to occur at an AUG codon. Near-cognate codons, such as CUG and ACG, can under certain circumstances also be recognized by Met–tRNA_i_. Initiation at a non-AUG codon normally requires a strong context (e.g. an A or G at −3 and a G at +4) and is enhanced when an RNA structure (e.g. a stem–loop) is able to form at a distance of approximately 14 nt 3′ of the initiation codon, so that it is positioned at the entrance of the mRNA channel when the potential initiation codon is in the P-site of the ribosome (Kozak, 1990; see also Clyde & Harris, 2006). The codons CUG, GUG, ACG, AUU, AUA, AUC and UUG are known to allow appreciable levels of initiation (e.g. 2–30 %), with CUG being the most efficient non-AUG initiation codon in many systems (reviewed by Touriol *et al.*, 2003). Non-AUG initiation may be widely used as a regulatory mechanism by cellular organisms (Ivanov *et al.*, 2008; Ingolia *et al.*, 2011) but, at present, it is unclear whether such regulatory aspects have relevance to the use of non-AUG initiation by RNA viruses.

As non-AUG initiation is nearly always relatively inefficient, an inevitable consequence is that a large proportion of ribosomes will scan past the non-AUG initiation site and initiate instead at downstream AUG codons or other near-cognate non-AUG codons. Thus, instances of non-AUG initiation in RNA viruses generally form part of a leaky-scanning mechanism for translating multiple N-terminal extension isoforms of a protein, or multiple proteins from alternative reading frames (Table 2). One of the first cases of non-AUG initiation described occurs in Sendai respirovirus (family *Paramyxoviridae*). An upstream in-frame ACG codon is used to initiate translation of C′, an N-terminally extended version of the C protein (Curran & Kolakofsky, 1988). C′ and C are encoded by an ORF that mostly overlaps the 5′ region of the P (phosphoprotein) coding sequence, with initiation codons in the order C′ (ACG), P (AUG), C (AUG). The AUG initiation codon for P lacks a purine at −3, thus all three proteins can be translated via leaky scanning. In the related virus, human parainfluenza respirovirus 1, C′, P and C proteins are produced, but here C′ translation initiates at a GUG codon with surprisingly high efficiency (Boeck *et al.*, 1992). An N-terminally extended version of the Gag polyprotein of murine leukemia gammaretrovirus has also been shown to initiate on an upstream in-frame non-AUG codon, in this case a CUG (Prats *et al.*, 1989). The N-terminal extension includes a signal peptide that directs the product to the endoplasmic reticulum. While the AUG-initiated Gag is the precursor of the virion structural proteins, the N-terminally extended version is not incorporated into virions, but undergoes glycosylation, is displayed on the surface of cells and plays a role in virus release (Nitta *et al.*, 2010). A homologous extension in feline leukemia gammaretrovirus initiates at an AUG codon with a weak context, thus potentially allowing leaky scanning to produce the N-terminally truncated isoform (Laprevotte *et al.*, 1984).

**Table 2.** Examples of known and suspected cases of non-AUG initiation

Taxon	Product	Initiation codon	References
*Respirovirus* – Sendai virus	C′ (N-term. extension of C)	ACG	Curran & Kolakofsky (1988); Gupta & Patwardhan (1988)
*Respirovirus* – human parainfluenza virus 1	"	GUG	Boeck *et al.* (1992)
Murine leukemia gammaretrovirus	Gag N-term. extension	CUG	Prats *et al.* (1989)
Soil-borne wheat mosaic furovirus and other furoviruses	CP N-term. extension	CUG	Shirako (1998)
*Panicovirus* – *Panicum* mosaic virus	Second movement protein of carmovirus-like double gene block	GUG	Turina *et al.* (2000)
*Panicovirus* – cocksfoot mild mosaic virus	"	CUG	Ziegler *et al.* (2009)
*Machlomovirus* – maize chlorotic mottle virus	"	CUG?	
*Pelargonium* line pattern virus	"	GUG	Castaño *et al.* (2009)
*Pelargonium* chlorotic ring pattern virus	"	GUG	
*Rosa rugosa* leaf distortion virus	"	CUG	
TGP carmovirus 3	"	CUG	Scheets *et al.* (2011)
TGP carmovirus 1	"	CUG/ACG?	
Allexiviruses and blackberry virus E	TGB3	CUG?	Kanyuka *et al.* (1992); Sabanadzovic *et al.* (2011)
Lily potexvirus X	TGB3	ACG?	Chen *et al.* (2005)
Strawberry mild yellow edge-associated potexvirus	TGB1	CUG?	Jelkmann *et al.* (1992)
Hibiscus chlorotic ringspot carmovirus	p27 (N-term. extension of p25)	CUG	Koh *et al.* (2006)
Aquareovirus A, segment 7	ORF1 (FAST protein)	CUG	Racine *et al.* (2009)
*Torovirus*	Predicted 30K protein	CUG	Firth & Atkins (2009)
Rice tungro bacilliform tungrovirus	ORF I	AUU	Fütterer *et al.* (1996)

**Fig. 3.**
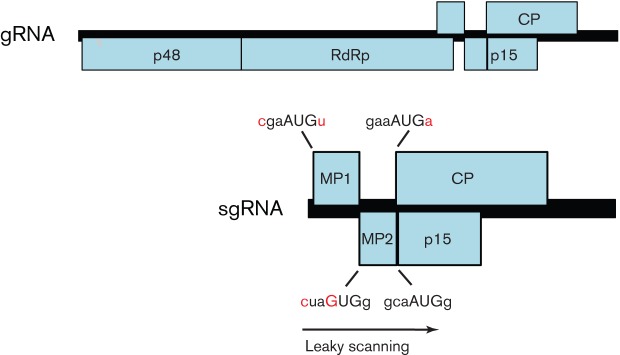
Genome map of *Panicum* mosaic panicovirus. MP1, MP2, CP and p15 are all expressed from a single sgRNA via a combination of leaky scanning and non-AUG initiation. Initiation codons are indicated in upper case and nucleotides that differ from a strong initiation context are indicated in red.

Many examples of non-AUG initiation come from plant viruses. In some cases, non-AUG initiation and leaky scanning are used to express three or even four separate proteins from a single transcript. One classical case, in rice tungro bacilliform tungrovirus (family *Caulimoviridae*), involves three consecutive ORFs where translation of the first ORF initiates at an AUU codon (facilitated by ribosome shunting; see below), the second ORF initiates at an AUG codon with a poor context, and the first two ORFs contain no other AUG codons, despite spanning around 895 nt, thus allowing leaky scanning also to the third ORF (Fütterer *et al.*, 1996, 1997). A second case that is looking increasingly widespread occurs in some members of the family *Tombusviridae*. Many viruses in this family produce two coding 3′-co-terminal sgRNAs – one to express the coat protein and another for expressing additional proteins from ORFs either 5′ or 3′ of the coat protein ORF. However, *Panicum* mosaic panicovirus produces only a single coding sgRNA from which four proteins, including the two carmovirus-like movement proteins, are expressed via a combination of non-AUG initiation and leaky scanning (Turina *et al.*, 2000; Fig. 3). The first movement protein, p8, has an AUG codon in a weak context; the second movement protein, p6.6 (which may be required in lower quantities; Li *et al.*, 1998), is expressed from a GUG initiation codon; and the coat protein and p15 (whose coding sequence overlaps the coat protein coding sequence) are expressed from AUG codons. Unusually, the GUG initiation codon is in a suboptimal context (cuaGUGg; cf. aacCUGg at the corresponding position in cocksfoot mild mosaic panicovirus). Maize chlorotic mottle machlomovirus and *Pelargonium* line pattern virus (proposed genus *Pelarspovirus*) also lack a separate sgRNA for coat protein expression and, like the panicoviruses, these and several related viruses also appear to use non-AUG initiation to express the second movement protein (Castaño *et al.*, 2009; Scheets *et al.*, 2011).

Another well-conserved but poorly appreciated case of non-AUG initiation appears to occur in the allexiviruses (family *Alphaflexiviridae*). Nearly all plant viruses encode one or more movement proteins that facilitate cell-to-cell movement through plasmodesmata. For many plant viruses, this takes the form of the ‘triple gene block’ – an evolutionarily conserved module that comprises three proteins, TGB1, TGB2 and TGB3 (Morozov & Solovyev, 2003). The TGBs are generally expressed from sgRNAs but TGB3 – which is required in much lower quantities than TGB2 – is normally translated inefficiently from the same sgRNA as TGB2 via leaky scanning (Verchot *et al.*, 1998; Morozov & Solovyev, 2003). While the allexiviruses clearly contain a TGB3 coding sequence, it lacks a suitable AUG initiation codon (Kanyuka *et al.*, 1992). Instead, in these viruses the TGB2 AUG initiation codon consistently has a weak context (C or U at −3, A at +4) thus facilitating leaky scanning, and TGB3 translation probably depends on non-AUG initiation. A conserved CUG codon in a strong context (A at −3, G at +4) provides one possible initiation site.

### Ribosome shunting

**Fig. 4.**
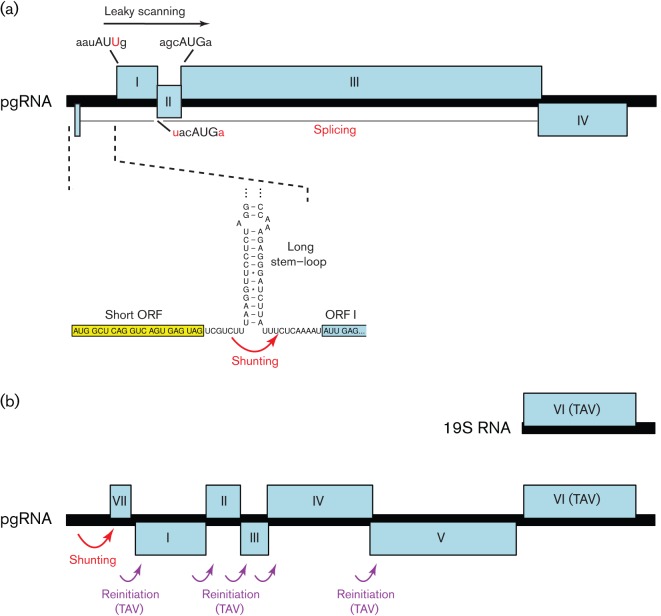
Ribosome shunting, reinitiation and leaky scanning in members of the family *Caulimoviridae*. (a) Translation of ORF I of the pgRNA of rice tungro bacilliform tungrovirus is by ribosome shunting. Here, 40S complexes released after translation of the short 5′-most ORF (yellow) are able to shunt past a stable stem–loop in the 5′UTR (red arrow) and continue scanning the mRNA. Reinitiation subsequently takes place at the start codons of either ORF I (a non-AUG codon, AUU), ORF II (weak context AUG) or ORF III (strong context AUG). ORF IV is expressed from a spliced mRNA. (b) In cauliflower mosaic caulimovirus, a similar shunting mechanism is used to access the 5′-most coding ORF, VII. However, downstream ORFs I–V are translated via reinitiation events that are stimulated by a viral reinitiation factor, transactivator viroplasmin (TAV), expressed from an sgRNA (see text).

While IRESes allow internal entry of ribosomes on a message in a 5′-end-independent manner, shunting allows ribosomes to access downstream ORFs in a manner that is 5′-end-dependent but, at least partly, scanning-independent. One of the best-studied examples occurs in viruses of the family *Caulimoviridae* – a family of plant-infecting pararetroviruses (Fütterer *et al.*, 1993; reviewed by Thiébeauld *et al.*, 2007). These viruses produce a longer-than-genome-length pregenomic RNA (pgRNA) from the circular genomic DNA. The pgRNA serves as the template for reverse transcription. Some members have 3′ ORFs that are translated from spliced or subgenomic RNAs. However, the pgRNA is generally polycistronic – containing several consecutive ORFs that are translated either via leaky scanning (in members of the genera *Tungrovirus* and *Badnavirus*; see above) or via reinitiation (in members of the genera *Caulimovirus* and *Soymovirus*; see below). The pgRNA is capped and has a long 5′UTR, much of which is predicted to fold into a large stem–loop structure. A short ORF terminates just upstream of the stem–loop (Fig. 4). Translation is 5′-cap-dependent and the 40S subunits of ribosomes that scan to and translate the short ORF are, upon termination, able to bypass the stem–loop (comprising e.g. approx. 480 nt including eight AUG codons in cauliflower mosaic caulimovirus) and resume scanning at a landing site just 3′ of the stem–loop (Schmidt-Puchta *et al.*, 1997; Pooggin *et al.*, 2006). It is thought that this ability depends on the small subunit of the ribosome retaining certain initiation factors during translation of the short ORF (see also section entitled Reinitiation), but that the (temporary) loss of other initiation factors promotes discontinuous scanning across the base of the stem–loop. The length and position of the short ORF, but not its sequence, are important for shunting and the short ORF must be translated for efficient shunting to occur (Hemmings-Mieszczak *et al.*, 2000; Pooggin *et al.*, 2006). The large stem–loop and 5′-adjacent short ORF are predicted to be present in most sequenced members of the family, suggesting that the shunt mechanism is a common feature of members of the *Caulimoviridae* (Pooggin *et al.*, 1999).

Shunting and/or partly scanning-independent mechanisms have also been proposed to explain non-canonical translation observed for the Y1 and Y2 proteins (N-terminally truncated versions of C) in Sendai respirovirus (de Breyne et al., 2004), the σC gene on segment S1 of avian orthoreovirus and Nelson Bay orthoreovirus (Racine & Duncan, 2010), the P (polymerase) gene of avihepadnaviruses (Cao & Tavis, 2011), and the *gag* gene of spumaviruses (Schepetilnikov *et al.*, 2009). Except for the last, these cases of shunting appear not to involve the short ORF and 3′-adjacent stem–loop configuration that occurs in members of the family *Caulimoviridae*.

### Reinitiation

After translation termination, the 40S and 60S subunits of the ribosome dissociate and, generally, both subunits leave the message. However, after translating a very short ORF (e.g. less than 30 codons), the 40S subunit may remain associated with the message, resume scanning, and reinitiate translation at a downstream AUG codon (reviewed by Jackson *et al.*, 2012). The ability of 40S subunits to remain associated with the message after translating a short ORF is thought to depend on certain initiation factors remaining attached to the ribosome during translation of the short ORF. After translation of the short ORF, the 40S subunit of the ribosome is not immediately competent to reinitiate, but becomes competent after scanning for some distance. This is thought to correspond to the time required to reacquire certain other initiation factors, including the eIF2–Met–tRNA_i_–GTP ternary complex. Such short ORFs are thought to be widely used in cellular genes to regulate expression of downstream protein-encoding ORFs in response to the cellular environment (Morris & Geballe, 2000; Ingolia *et al.*, 2011). In contrast, reinitiation after translation of a long ORF – e.g. one that encodes a functional product – is much rarer and requires special signals within the mRNA or specific *trans*-acting protein factors (reviewed by Thiébeauld *et al.*, 2007; Powell, 2010; Jackson *et al.*, 2012). A number of distinct mechanisms appear to be used by different viruses (Table 3).

**Table 3.** Examples of known and suspected cases of reinitiation

Taxon	Products	References
*Caliciviridae* – *Lagovirus*, *Vesivirus*, *Norovirus*, *Sapovirus*, *Nebovirus*	VP1/VP2	Meyers (2003); Luttermann & Meyers (2007); Napthine *et al.* (2009)
*Caliciviridae* – some noroviruses	Replicase/VP1	McCormick *et al.* (2008)
*Orthomyxoviridae* – influenza virus B, segment 7	M1/BM2	Horvath *et al.* (1990); Powell *et al.* (2008); Powell *et al.* (2011)
*Pneumovirinae* – *Pneumovirus*, *Metapneumovirus*	M2-1/M2-2	Ahmadian *et al.* (2000); Gould & Easton (2005); Gould & Easton (2007)
*Totiviridae* – *Victorivirus*	Gag/Pol	Huang & Ghabrial (1996); Soldevila & Ghabrial (2000); Ghabrial & Nibert (2009); Li *et al.* (2011)
*Hypovirus* – *Cryphonectria* hypoviruses 1 and 2	ORFA/ORFB	Shapira *et al.* (1991); Hillman *et al.* (1994); Guo *et al.* (2009)
*Caulimoviridae* – *Caulimovirus*, *Soymovirus*	Multiple consecutive ORFs	Fütterer & Hohn (1991); Scholthof *et al.* (1992); Maiti *et al.* (1998)

**Fig. 5.**
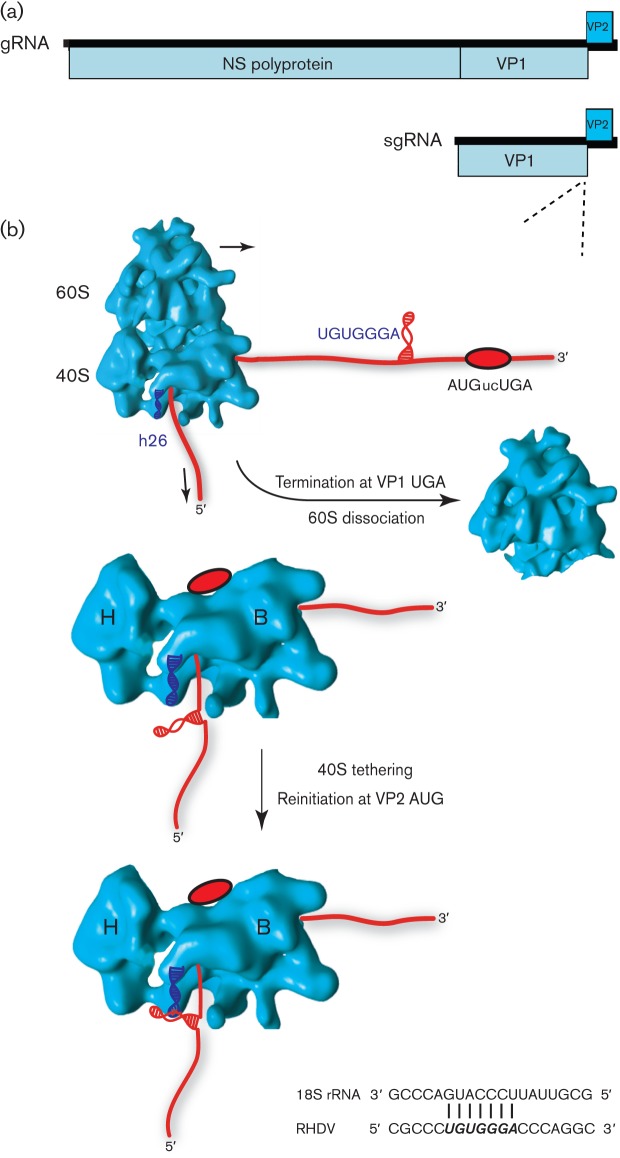
Proposed model for termination–reinitiation in caliciviruses. (a) Genome map of the calicivirus rabbit hemorrhagic disease lagovirus (RHDV). Expression of VP2 is by termination–reinitiation during translation of the viral sgRNA. (b) As the ribosome approaches the termination–reinitiation site (red oval; AUGucUGA in RHDV), the stretch of RNA containing TURBS motif 1 (UGUGGGA), predicted to be located in an RNA secondary structure, is translated and may be remodelled. During termination, the secondary structure is located in the mRNA exit channel of the ribosome [located between the head (H) and body (B) of the 40S subunit] and in close proximity to the solvent-accessible helix 26 (h26) of 18S rRNA (indicated as a blue helix). Base pairing between complementary residues in motif 1 and h26 occurs (shown at the bottom), with the interaction likely to be stabilized by eIF3 (not shown), also known to contact the TURBS and 18S rRNA. Together, these interactions act to tether the ribosome to the viral RNA, preventing its dissociation, allowing time for the recruitment of initiation factors and subsequent reinitiation on the downstream VP2 ORF.

Calicivirus genomes generally contain at least three protein-encoding ORFs: ORF1 is translated from the genomic RNA and encodes the non-structural proteins, whilst ORF2 and ORF3 are translated from a single sgRNA and encode, respectively, the major capsid protein and a small basic protein that is a minor component of the virion (Herbert *et al.*, 1996). In some genera (e.g. *Lagovirus* and *Sapovirus*), ORF2 is contiguous with ORF1 so that some capsid protein is also translated from the genomic RNA as a fusion with the non-structural polyprotein, even though the major source of capsid protein translation is still the sgRNA (Fig. 5). ORF3 is positioned at the 3′ end of the sgRNA such that its initiation codon is located very close to the ORF2 termination codon (often AUGnnUGA or overlapping as UAAUG or AUGA), and is translated via reinitiation after translation of ORF2 (Meyers, 2003; Luttermann & Meyers, 2007; Pöyry *et al.*, 2007; Napthine *et al.*, 2009). Here, reinitiation is dependent on RNA sequence motifs typically within the 40–90 nucleotides directly upstream of the ORF2 termination codon. This sequence region is termed the TURBS (termination upstream ribosome-binding site) and contains a short sequence motif (motif 1; UGGGA and flanking nucleotides) that is complementary to the loop region of helix 26 of 18S rRNA (the RNA component of the 40S subunit). Interaction between motif 1 and 18S rRNA has been shown to be required for efficient reinitiation in a yeast system in which 18S rRNA could be mutated (Luttermann & Meyers, 2009). The TURBS has also been shown to bind eIF3 (Pöyry *et al.*, 2007). It is thought that a proportion of 40S subunits of ribosomes terminating translation of ORF2 are tethered with eIF3 to the mRNA via interaction with the TURBS and, following recruitment of the eIF2–Met–tRNA_i_–GTP ternary complex, such subunits may subsequently initiate translation of ORF3 (Fig. 5). Notably, and in contrast to reinitiation after a very short ORF, eIF4G is not required (Pöyry *et al.*, 2007). A similar reinitiation mechanism also appears to be used by influenza B virus for translation of the BM2 protein (Horvath *et al.*, 1990). Here, the M1 and BM2 coding sequences overlap with the sequence UAAUG and an upstream TURBS, comprising around 45 nt upstream of the termination codon and incorporating an appropriately positioned UGGGA motif, is again crucial for reinitiation (Powell *et al.*, 2011). Mutational analyses have demonstrated that increasing the distance between the TURBS and the termination codon reduces reinitiation efficiency, presumably due to reduced tethering of post-termination 40S subunits, but reinitiation still preferentially occurs at the natural position with respect to the TURBS (Pöyry *et al.*, 2007; Powell *et al.*, 2011). In some cases, initiation codons some distance downstream of the natural reinitiation site may be utilized at reduced efficiency if the natural reinitiation site is mutated (Powell *et al.*, 2011). Non-AUG initiation codons may also be utilized, although AUG codons are preferred if available (Luttermann & Meyers, 2007; Pöyry *et al.*, 2007).

Interestingly, despite the production of an sgRNA for ORF2 expression, some noroviruses are also capable of translating ORF2 via reinitiation after translation of ORF1 from the genomic RNA (McCormick *et al.*, 2008). Again, this reinitiation is dependent on an upstream TURBS. As certain other caliciviruses are also capable of expressing the capsid protein from the genomic RNA due to ORF2 being contiguous with ORF1 (see above), it has been hypothesized that the capsid protein may play additional roles early in virus infection before sgRNAs are produced (McCormick *et al.*, 2008).

A termination–reinitiation mechanism is also used by pneumoviruses and metapneumoviruses to express their M2-1 and M2-2 proteins from a single transcript (Ahmadian *et al.*, 2000; Gould & Easton, 2007). As with caliciviruses, reinitiation is dependent on sequences upstream of the termination codon, but a much larger sequence region is involved (e.g. approx. 250 nt are required to achieve 80 % of the wild-type reinitiation efficiency in respiratory syncytial pneumovirus) and a TURBS-like sequence has not been identified. Interestingly, the reinitiation AUG codon can be some distance upstream of the termination codon (e.g. up to 29 nt in respiratory syncytial pneumovirus, although two other AUG codons that are closer to the termination codon are also utilized; Ahmadian *et al.*, 2000). In members of the genus *Victorivirus* of the family *Totiviridae*, reinitiation after translation of the capsid (Gag) coding sequence is used to translate the polymerase (Pol) – which is required in much lower quantities – from the single genomic RNA (Li *et al.*, 2011). The two ORFs often overlap with AUGA or sometimes AUGnnUAG or UAGnnAUG. Again, upstream sequences – e.g. at least 32 nt in *Helminthosporium victoriae* victorivirus 190S – have been shown to be important for reinitiation (Li *et al.*, 2011).

Whilst reinitiation in the above viruses is mediated partly or wholly by RNA sequences directly upstream of the termination codon, a very different reinitiation mechanism is used by members of the genera *Caulimovirus* and *Soymovirus* in the family *Caulimoviridae* (see section entitled Ribosome shunting). Here, reinitiation is mediated by a viral protein ‘transactivator viroplasmin’ or TAV (Fütterer & Hohn, 1991; Scholthof *et al.*, 1992; reviewed by Thiébeauld *et al.*, 2007). In cauliflower mosaic caulimovirus, TAV is encoded by ORF VI, which is translated from a subgenomic 19S transcript, while several other consecutive ORFs are translated from the pgRNA via TAV-mediated reinitiation (Fig. 4). TAV-mediated reinitiation does not appear to require any specific sequence elements in the RNA transcript (Fütterer & Hohn, 1991). Through interactions with the host proteins TOR and RISP, TAV is thought to tether eIF3 to the elongating ribosome during translation of the upstream ORF, thus assisting reinitiation on downstream ORFs (Schepetilnikov *et al.*, 2011).

## Non-canonical elongation and termination

Non-canonical translation mechanisms that act during elongation, or via altered readings of termination signals, are known as recoding mechanisms (reviewed by Atkins & Gesteland, 2010). These fall into four main categories: ribosomal frameshifting, whereby a proportion of ribosomes are directed into a different reading frame by shifting forwards or backwards 1 or 2 nt; bypassing, in which a proportion of ribosomes skip over a larger number of nucleotides and continue translating; stop-codon redefinition and readthrough, whereby a proportion of ribosomes fail to terminate at a stop codon but instead insert a standard amino acid (readthrough) or a non-standard amino acid such as selenocysteine or pyrrolysine (redefinition); and stop–carry on – a mechanism that directs co-translational separation of the peptide chain by preventing peptide-bond formation at a specific site. Programmed bypassing and selenocysteine/pyrrolysine insertion are not, however, known to be utilized by eukaryote-infecting RNA viruses.

### Ribosomal frameshifting

**Fig. 6.**
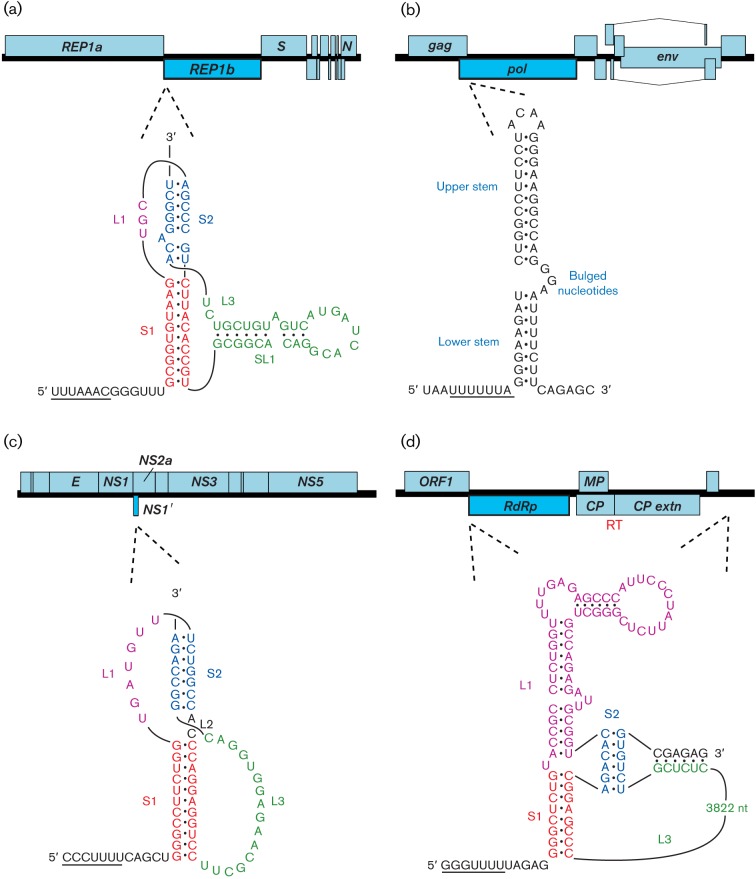
Programmed ribosomal frameshifting in viral mRNAs. In each of the four examples, the genome is indicated as an ORF map, with the location of the frameshift site shown by dotted lines. In SARS coronavirus (a), Japanese encephalitis flavivirus (c) and barley yellow dwarf luteovirus (d), frameshifting is stimulated by an RNA pseudoknot (including a long-range interaction in the luteovirus). In HIV-1 (b), frameshifting is stimulated by a two-stem helix, although the upper stem makes the major contribution to frameshifting efficiency. In each case, the slippery shift site sequence is underlined. Note that, in (c), the unprocessed frameshift product generates a truncated polyprotein, unlike the other examples, where the frameshift facilitates extension of the polyprotein. Spliced and subgenomic RNAs are not shown and polyprotein cleavage products are only indicated where specifically relevant. ‘RT’ indicates a stop codon readthrough site.

Programmed −1 ribosomal frameshifting was first described as the mechanism by which the Gag–Pol polyprotein of Rous sarcoma alpharetrovirus is expressed from overlapping *gag* and *pol* ORFs (Jacks & Varmus, 1985; Jacks *et al.*, 1988). Related signals have since been documented in many other viruses (see Table 4), including the clinically important lentiviruses HIV-1 and HIV-2, human T-cell lymphotrophic deltaretrovirus types 1 and 2, and the coronavirus responsible for severe acute respiratory syndrome (SARS-CoV). Frameshifting has also been increasingly recognized in conventional cellular genes of both prokaryotes and eukaryotes, as well as in other replicating elements, such as insertion sequences and transposons (reviewed by Giedroc & Cornish, 2009; Brierley *et al.*, 2010). The mRNA signal for −1 frameshifting in eukaryotic systems comprises two elements: a slippery sequence with consensus X_XXY_YYZ (underlines separate zero-frame codons; XXX represents any three identical nucleotides, YYY represents AAA or UUU, and Z represents A, C or U) where the ribosome changes frame, and a downstream stimulatory RNA structure (Fig. 6; reviewed by Giedroc & Cornish, 2009; Brierley *et al.*, 2010). Appropriate spacing (typically 5–9 nt) between the slippery sequence and the stimulatory RNA is also required for efficient frameshifting.

**Table 4.** Examples of known and suspected cases of programmed −1 ribosomal frameshifting

Taxon	Product	References
*Astroviridae* – *Avastrovirus*, *Mamastrovirus*	Replicase	Marczinke *et al.* (1994)
*Arteriviridae* – *Arterivirus*	Replicase	den Boon *et al.* (1991)
*Coronaviridae* – *Alphacoronavirus*, *Betacoronavirus*, *Gammacoronavirus*, *Bafinivirus*, *Torovirus*	Replicase	Brierley *et al.* (1987); Bredenbeek *et al.* (1990); Herold & Siddell (1993); Snijder *et al.* (1990); Thiel *et al.* (2003)
*Roniviridae* – *Okavirus*	Replicase	Cowley *et al.* (2000)
Unclassified *Nidovirales* – Nam Dinh virus, Cavally virus	Replicase	Nga *et al.* (2011)
*Sobemovirus*	Replicase	Mäkinen *et al.* (1995)
*Barnaviridae* – *Barnavirus*	Replicase	Revill *et al.* (1994)
*Polemovirus*	Replicase	aus dem Siepen *et al.* (2005)
*Luteoviridae* – *Polerovirus*, *Enamovirus*	Replicase	Demler & de Zoeten (1991); Prüfer *et al.* (1992); Garcia *et al.* (1993)
*Luteoviridae* – *Luteovirus*	Replicase	Brault & Miller (1992)
*Umbravirus*	Replicase	Demler *et al.* (1993); Gibbs *et al.* (1996)
*Tombusviridae* – *Dianthovirus*	Replicase	Xiong *et al.* (1993); Kim & Lommel (1994); Tajima *et al.* (2011)
*Totiviridae* – *Trichomonasvirus* (*Trichomonas vaginalis* viruses 2, 3 and 4)	Replicase	Bessarab *et al.* (2000); Bessarab *et al.* (2011)
*Totiviridae* – *Totivirus* (*Saccharomyces cerevisiae* viruses L–A and L–BC, *Tuber aestivum* virus 1, black raspberry virus F)	Replicase	Diamond *et al.* (1989); Dinman *et al.* (1991)
*Totiviridae* – *Giardiavirus*	Replicase	Wang *et al.* (1993); Li *et al.* (2001)
*Totiviridae* – ‘*Artivirus*, (penaeid shrimp infectious myonecrosis virus, *Armigeres subalbatus* virus, *Drosophila melanogaster* totivirus, Omono River virus)	Replicase	Nibert (2007); Zhai *et al.* (2010); Wu *et al.* (2010); Isawa *et al.* (2011)
Unclassified *Totiviridae* (*Lentinula edodes* mycovirus, *Phlebiopsis gigantea* mycovirus 1, *Phlebiopsis gigantea* mycovirus 2, *Fusarium graminearum* mycovirus 3, piscine myocarditis virus, grapevine associated totivirus 2)	Replicase	Ohta *et al.* (2008); Kozlakidis *et al.* (2009); Yu *et al.* (2009); Haugland *et al.* (2011); Al Rwahnih *et al.* (2011)
Unclassified dsRNA viruses (*Rosellinia necatrix* megabirnavirus, *Spissistilus festinus* virus 1, *Circulifer tenellus* virus 1)	Replicase	Chiba *et al.* (2009); Spear *et al.* (2010)
*Retroviridae* – *Lentivirus*, *Alpharetrovirus*	Reverse transcriptase (Gag–Pol)	Jacks & Varmus (1985); Jacks *et al.* (1988a); Jacks *et al.* (1988b); Morikawa & Bishop (1992)
*Retroviridae* – *Betaretrovirus*, *Deltaretrovirus*	Reverse transcriptase (Gag–Pro–Pol)	Moore *et al.* (1987); Jacks *et al.* (1987); Nam *et al.* (1988); Mador *et al.* (1989); Nam *et al.* (1993)
Unclassified ssRNA+ viruses – *Acyrthosiphon pisum* virus, rosy apple aphid virus	CP-extension	van der Wilk *et al.* (1997)
*Togaviridae* – *Alphavirus*	TF	Firth *et al.* (2008); Chung *et al.* (2010)
*Flaviviridae* – *Flavivirus* (Japanese encephalitis serogroup)	NS1′	Firth & Atkins (2009); Melian *et al.* (2010)
*Flaviviridae* – *Flavivirus* (insect-specific flaviviruses)	FIFO	Firth *et al.* (2010)
*Picornaviridae* – *Cardiovirus*	2B*	Loughran *et al.* (2011)

There is considerable experimental support for the idea that ‘tandem slippage’ of ribosome-bound peptidyl– and aminoacyl–tRNAs on the slippery sequence occurs upon encounter of the stimulatory RNA, with the tRNAs detaching from the zero-frame codons (XXY_YYZ) and re-pairing in the −1 frame (XXX_YYY), thus allowing for perfect re-pairing except at the wobble positions (Jacks *et al.*, 1988). As the codon : anticodon duplex in the P-site is not monitored as strictly as that in the A-site (Ogle *et al.*, 2001), certain deviations from the canonical XXX of the slippery site are tolerated, including GUU in equine arteritis arterivirus, GGA in insect-specific flaviviruses, and GGU in encephalomyocarditis cardiovirus. The stimulatory RNA generally takes the form of a stem–loop or RNA pseudoknot and, in most cases, is a discrete local element. However, some 3′ stimulatory structures have an additional long-range base-pairing component that may provide a regulatory link between translation and replication (e.g. barley yellow dwarf luteovirus; Barry & Miller, 2002). How the stimulatory RNAs function to promote frameshifting is still uncertain, but accumulating evidence implicates the intrinsic unwinding activity of the ribosome (Qu *et al.*, 2011), with the stimulatory RNA exhibiting resistance to unwinding, perhaps by presenting an unusual topology (Plant & Dinman, 2005; Namy *et al.*, 2006). Failure to unwind the stimulatory RNA appropriately has been proposed to induce tension in the mRNA, leading to uncoupling of the codon : anticodon complexes and realignment of the tRNAs in the −1 frame (Plant & Dinman, 2005; Namy *et al.*, 2006).

Many positive-strand RNA viruses, most retroviruses, and some members of the family *Totiviridae* of dsRNA viruses make use of −1 frameshifting to express their RdRp or reverse transcriptase (Table 4). Indeed, in the case of members of the genera *Betaretrovirus* and *Deltaretrovirus*, where the protease gene (*pro*) is encoded as a separate ORF, two frameshifts are required to express the Gag–Pro–Pol polyprotein. There are numerous potential advantages in using frameshifting as an expression strategy. In retroviruses and totiviruses, for example, it allows the virus to generate a defined ratio of Gag : Gag–Pol that is likely to be optimized for virion assembly and allows facile targeting of the replicative enzymes to the virion core. It also obviates the need to produce a separate mRNA for expression of the viral polymerase. Similarly, in many positive-strand RNA viruses, frameshifting may serve to produce the polymerase at a fixed ratio relative to other components of the replication complex (reviewed by Ahlquist, 2006). Indeed, artificially altering the frameshifting efficiency has proven to be attenuating in several cases (Dulude *et al.*, 2006; Plant *et al.*, 2010), although in HIV-1 there is evidence that a modest stimulation of frameshifting can actually increase infectivity (Miyauchi *et al.*, 2006).

Frameshifting is also utilized for the expression of proteins unrelated to polymerases. In *Acyrthosiphon pisum* virus, an unclassified virus that is related distantly to viruses of the family *Picornaviridae*, −1 frameshifting at the end of a long polyprotein-encoding ORF1 provides access to a 3′-terminal ORF2 (van der Wilk *et al.*, 1997). Here, the 3′ end of ORF1 encodes the major virion protein (34K), while the transframe fusion gives rise to a minor virion protein (66K). In alphaviruses, the Japanese encephalitis serogroup of flaviviruses, and the cardioviruses, −1 frameshifting provides access to short ORFs overlapping internal regions of long polyprotein-encoding ORFs to generate transframe proteins that are N-terminally coincident with one of the polyprotein cleavage products (Firth *et al.*, 2008; Melian *et al.*, 2010; Loughran *et al.*, 2011). In the Japanese encephalitis serogroup, frameshifting adds a 52 aa transframe C-terminal extension to the NS1 protein to produce the NS1′ protein. In the alphaviruses, frameshifting produces an 8 kDa protein that shares the N-terminal approximately two-thirds with the 6K protein, but has a hydrophilic instead of a hydrophobic C-terminal region. In encephalomyocarditis cardiovirus, frameshifting produces a 128 or 129 aa protein, 2B*, in which just the N-terminal 11–12 aa are encoded by the zero frame.

In a few taxa, +1 (or −2) frameshifting appears to be utilized to express the viral polymerase. However, in viruses these mechanisms have been far less well-studied than −1 tandem slippage and remain poorly understood, in part because the frameshift efficiencies, where investigated, often seem to be very low. In members of the *Closteroviridae* – a family of large positive-strand plant-infecting RNA viruses – the RdRp is encoded by ORF2, which is in the +1 frame relative to ORF1, while many 3′ ORFs are translated from sgRNAs. As the RdRp is required to produce sgRNAs, it was proposed that ORF2 is translated via +1 frameshifting at or near the end of ORF1 (Agranovsky *et al.*, 1994). Indeed now, with many highly divergent genome sequences available, it is clear from comparative sequence analysis that mechanisms involving AUG-initiation cannot, in general, mediate ORF2 translation. However the exact frameshifting mechanism remains something of a mystery. In many closteroviruses, frameshifting is thought to occur on a highly conserved GUU_stop_C motif at the ORF1 stop codon and may involve +1 nt P-site slippage from GUU to UUU, with the slow-to-decode stop codon in the A-site. In citrus tristeza closterovirus, however, frameshifting appears to occur upstream of the ORF1 stop codon (as evidenced by a conserved overlap region and high conservation at ORF1-frame synonymous sites for at least 25 codons upstream of the ORF1 stop codon), and frameshifting has been suggested to occur on a GUU_CGG_C sequence that aligns with the GUU_stop_C sequence in other closteroviruses (Karasev *et al.*, 1995; but compare with Çevik, 2001).

Whilst many members of the family *Totiviridae* utilize −1 tandem slippage to express a Gag–Pol fusion, or reinitiation to express Gag and Pol separately, a few members apparently utilize +1 or −2 frameshifting. *Trichomonas vaginalis* virus 1 (genus *Trichomonasvirus*) is particularly interesting. Here, ORF2 is in the +1 frame relative to ORF1 and nucleotide sequence analysis indicates that frameshifting is most likely to occur on a conserved CC_CUU_UUU sequence (Su & Tai, 1996; Goodman *et al.*, 2011). Notably, the 5′ CC is conserved despite the corresponding ORF1-frame xCC codon being GCC, UCC or ACC in different isolates, suggesting that frameshifting is by −2 nt and not +1 nt. In contrast, in *Trichomonas vaginalis* viruses 2, 3 and 4, ORF2 is in the −1 frame relative to ORF1, and −1 frameshifting is predicted to occur on a G_GGC_CCY heptanucleotide – a sequence that, due to the C_CCY A-site tetranucleotide, is not usually associated with efficient frameshifting, but which may nevertheless be adequate for the low level of frameshifting that is apparently required by these viruses.

The *Amalgamaviridae* – a recently proposed family of monopartite dsRNA viruses (Martin *et al.*, 2011), besides *Leishmania* RNA virus 1 (genus *Leishmaniavirus*, family *Totiviridae*; Kim *et al.*, 2005), and the unclassified positive-strand RNA viruses chronic bee paralysis virus and Lake Sinai viruses 1 and 2 (Olivier *et al.*, 2008), also appear to require +1 slippage to express their RdRp, although the shift sites have not yet been definitively localized nor have alternative mechanisms been ruled out.

Recently, a short conserved ORF that overlaps the P3-encoding region of the polyprotein ORF in probably all members of the *Potyviridae* (the largest family of RNA plant viruses) was shown to be translated and essential for virus infectivity (Chung *et al.*, 2008; Wen & Hajimorad, 2010; Wei *et al.*, 2010; for earlier insights see also Gibbs & Keese, 1995; Choi *et al.*, 2001). In turnip mosaic potyvirus, the ORF (known as *pipo*) is expressed as part of an approximately 25 kDa product that is believed to correspond to a fusion of PIPO with the N-terminal region of P3 (i.e. P3N–PIPO). The frameshifting mechanism has not yet been determined, but current evidence suggests that it occurs at the level of translation rather than transcription, and most likely involves a highly conserved GAA_AAA_A motif at the 5′ end of the *pipo* ORF (Chung *et al.*, 2008). Notably, the GAA_AAA_A motif is in a different frame relative to the canonical X_XXY_YYZ −1 tandem slippage site, suggesting a +2 rather than a −1 shift.

### Stop-codon readthrough

Translation termination is generally a highly efficient process, but is influenced by the nature of the stop codon present (UAA, UAG or UGA) and its flanking nucleotides, especially the immediately adjacent 3′ base (reviewed by Bertram *et al.*, 2001). Some termination codon contexts are noticeably ‘leaky’ (e.g. UGAC; McCaughan *et al.*, 1995), i.e. they allow ‘readthrough’ at frequencies ranging from 0.3 to 5 % (Bertram *et al.*, 2001). In readthrough, the stop codon is decoded by a near-cognate or suppressor tRNA, and translation continues to the next termination codon. Readthrough is exploited in the expression of several viral and cellular genes, where it is referred to as programmed readthrough (reviewed by Beier & Grimm, 2001; Namy & Rousset, 2010). Readthrough allows the production of a C-terminally extended polypeptide at a defined frequency. In viruses, it is often used to express the polymerase, but another common use is to append an extension domain to a proportion of coat proteins. In the luteoviruses, for example, readthrough at the end of the coat protein gene generates a protein required for aphid transmission (Brault *et al.*, 1995). Similarly, in benyviruses and pomoviruses the coat protein extension is required for transmission by their plasmodiophorid vectors (reviewed by Adams *et al.*, 2001).

The efficiency of readthrough can be influenced by elements located both 5′ and 3′ of the suppressed stop codon. Adenosines in the two positions immediately preceding the stop codon have been shown to stimulate readthrough (albeit in a yeast-cell environment) and are a feature common to many viral readthrough cases – notably in the tobamoviruses, poleroviruses and luteoviruses (Beier & Grimm, 2001; Tork *et al.*, 2004). Downstream stimulators generally fall into two classes: the 3′-adjacent nucleotides, which are thought to act at the level of primary sequence, and more distal elements that typically involve RNA secondary structures. The effect of the immediately 3′-adjacent nucleotide(s) may be specific to the identity of the stop codon (Bonetti *et al.*, 1995), and this may relate to competition between the release factor and potential near-cognate tRNAs binding to the stop codon. In contrast, 3′ RNA structures typically beginning around 8 nt 3′ of the stop codon are a common (but not ubiquitous) feature of different types of readthrough. The exact mechanism(s) by which such structures promote readthrough is not currently known, but possibilities include modulation of ribosome activity directly through mRNA–protein or mRNA–rRNA interactions; interference with release factor function through steric hindrance; or, similar to frameshift stimulatory RNAs, by providing a barrier to unwinding by a ribosome-associated helicase (Qu *et al.*, 2011; although at least some readthrough-stimulating structures do not, on their own, cause significant ribosome pausing; Napthine *et al.*, 2012).

**Fig. 7.**
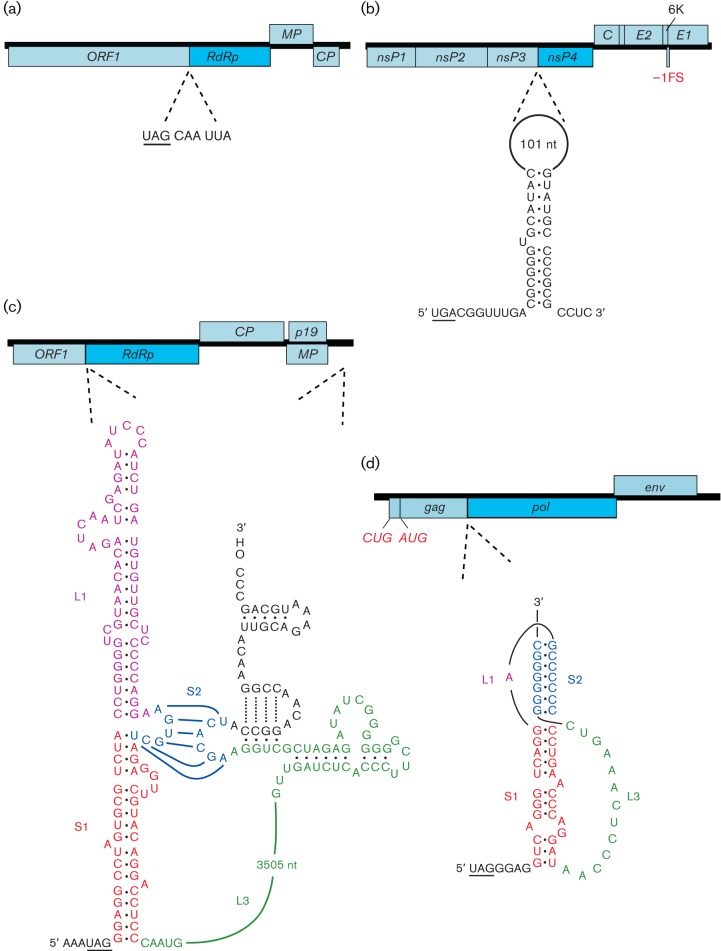
Programmed stop codon readthrough in viral mRNAs. In each of the four examples, the genome is indicated as an ORF map, with the location of the readthrough site shown by dotted lines. In tobacco mosaic tobamovirus (a), only a short local sequence context 3′ of the recoded UAG is required for efficient readthrough. In Venezuelan equine encephalitis alphavirus (b), carnation italian ringspot tombusvirus (c) and murine leukemia gammaretrovirus (d), the 3′ stimulator is an RNA secondary structure: an extended stem–loop in (b), an RNA pseudoknot in (d) and long-range base pairing in (c). Spliced and subgenomic RNAs are not shown and polyprotein cleavage products are only indicated where specifically relevant. ‘FS’ indicates a ribosomal frameshift site.

The various 3′ motifs that stimulate readthrough in eukaryote-infecting viruses have been divided into three broad classes (Beier & Grimm, 2001; Fig. 7; Table 5). The type I motif is exemplified by tobacco mosaic virus and other tobamoviruses. Here, readthrough of a UAG codon in the replicase gene is stimulated by the six nucleotides immediately following the stop codon, with the consensus motif for efficient readthrough being UAG_CAR_YYA (Skuzeski *et al.*, 1991; R = purine, Y = pyrimidine). The same motif is utilized by a number of other plant viruses; for example in benyviruses and pomoviruses, where readthrough generates extended versions of the viral coat protein. Although natural cases of CARYYA-stimulated readthrough generally involve a UAG codon, CARYYA can also stimulate readthrough of UGA and UAA codons (Skuzeski *et al.*, 1991). The type II motif was originally defined as generally comprising a UGA stop codon followed by a CGG or CUA triplet (Beier & Grimm, 2001). It was later proposed that most instances of readthrough in this class also involve a 3′ RNA structure component – often comprising an extended stem–loop structure beginning around 8 nt 3′ of the stop codon (Firth *et al.*, 2011; Napthine *et al.*, 2012). Type II readthrough occurs in the replicase gene of a number of alphaviruses (although not all alphaviruses have an internal stop codon in their replicase gene), the replicase gene of tobraviruses, pecluviruses, furoviruses and pomoviruses, the coat protein extension gene of furoviruses, and the VP9/VP9′ gene of coltiviruses.

**Table 5.** Examples of known and suspected cases of stop-codon readthrough

Taxon	Product	Type	Stop-codon context	RNA structure	References
*Alphavirus* (species that utilize readthrough)	Replicase	II	**UGA**-CGG, **UGA**-CUA	Extended stem–loop	Li & Rice (1993); Firth *et al.* (2011)
*Tobravirus* RNA1	Replicase	II	**UGA**-CGG	Extended stem–loop*	Urban *et al.* (1996); Firth *et al.* (2011)
*Pecluvirus* RNA1	Replicase	II	**UGA**-CGG	Extended stem–loop?	
*Furovirus* RNA1	Replicase	II	**UGA**-CGG	Extended stem–loop?	
*Pomovirus* RNA1	Replicase	II	**UGA**-CGG, **UAA**-CGG etc.	Extended stem–loop*	
*Tobamovirus*	Replicase	I	A-**UAG**-CAA-UUA		Skuzeski *et al.* (1991); Zerfass & Beier (1992)
Providence virus	Replicase	I	**UAG**-CAA-CUA		Walter *et al.* (2010)
*Tombusviridae* (except *Dianthovirus*)	Replicase	III	**UAG**-GGR	3′-proximal structure+long-distance 3′ base pairing	Cimino *et al.* (2011)
*Gammaretrovirus*	Reverse transcriptase	III	**UAG**-G	Compact pseudoknot	Wills *et al.* (1991); Alam *et al.* (1999)
*Epsilonretrovirus*	Reverse transcriptase	III	**UAG**-R	Extended stem–loop†	
*Furovirus* RNA2	CP-extension	II	**UGA**-CGG etc.	Extended stem–loop?	
*Pomovirus* RNA2	CP-extension	I	**UAG**-CAA-UYA, **UAA**-CAA-UUA		
*Luteovirus*, *Polerovirus*	CP-extension	III	AAA-**UAG**-GUA	Long-distance 3′ base pairing?	Brown *et al.* (1996); Bruyère *et al.* (1997)
Rose spring dwarf-associated luteovirus	CP-extension	II	**UGA**-CGG		
*Enamovirus*	CP-extension		**UGA**-GGG		Demler & de Zoeten (1991)
*Benyvirus*	CP-extension	I	**UAG**-CAA-UUA	Compact stem–loop*	
Rice stripe necrosis benyvirus	CP-extension	III	**UAG**-GGG	Compact stem–loop*	
*Coltivirus*, segment 9	VP9′	II	**UGA**-CGG	Extended stem–loop	Jaafar *et al.* (2004); Napthine *et al.* (2012)

* R. Ling & A. E. Firth, unpublished data.

† S. Napthine, K. E. Deigan & I. Brierley, unpublished data.

Type III readthrough motifs comprise a more diverse class, but generally involve a UAG stop codon, a 3′-adjacent G or purine-rich octanucleotide, and some form of 3′ RNA structure. In murine leukemia virus and other gammaretroviruses, efficient readthrough of a UAG codon in the replicase gene requires a compact 3′-adjacent pseudoknot structure, with the identity of certain nucleotides in the 8 nt ‘spacer’ region between the stop codon and the pseudoknot also being important (Alam *et al.*, 1999; Houck-Loomis *et al.*, 2011). In luteoviruses and poleroviruses, efficient readthrough of a UAG codon is dependent on 3′-adjacent sequences, but an element located approximately 700–750 nt 3′ of the stop codon is also important, and long-distance RNA base pairing between the 3′-proximal and 3′-distal elements has been suggested as a possible mechanism (Brown *et al.*, 1996). In members of the family *Tombusviridae*, such as carnation italian ringspot tombusvirus and turnip crinkle carmovirus, efficient readthrough is dependent on a large 3′-proximal RNA stem–loop structure, besides long-distance RNA base pairing between this structure and an element near the 3′ end of the genome, 3.5 kb away (Cimino *et al.*, 2011). Similar long-distance interactions have been predicted for other genera** in the family *Tombusviridae*, including *Necrovirus*, *Aureusvirus* and *Panicovirus*, and it has been proposed that the long-distance interaction may play a regulatory role by linking translation (of the RdRp) with replication (Cimino *et al.*, 2011).

### Stop–carry on

Stop–carry on is mediated by the amino acid motif D(V/I)ExNPGP, which, together with less-conserved but nonetheless functionally important upstream amino acids also within the ribosome exit tunnel (approx. 30 aa in total), prevents formation of a peptide bond between glycine and the final proline (Donnelly *et al.*, 2001; Doronina *et al.*, 2008; reviewed by Brown & Ryan, 2010). Nonetheless, translation can continue (with proline as the N-terminal amino acid of the downstream product) with up to near-100 % efficiency. It is thought that the structure of the nascent peptide within the ribosome prevents Pro–tRNA from binding in the A-site, but instead allows eRF1 to bind and, with eRF3, release the nascent peptide, following which Pro–tRNA is able to bind and translation proceeds. As such, stop–carry on provides an alternative mechanism to proteolytic cleavage for producing multiple protein products from a single ORF. Stop–carry on cassettes are present in diverse RNA viruses and have probably evolved (or been acquired) independently on more than one occasion. One of the most well-known occurrences is at the junction between the 2A and 2B proteins of members of several genera of the family *Picornaviridae*, including the aphthoviruses, cardioviruses, erboviruses and teschoviruses. Stop–carry on cassettes also occur in (some) members of the taxa *Iflavirus*, *Dicistroviridae*, *Tetraviridae*, *Rotavirus*, *Cypovirus* and *Totiviridae*, with some viruses having multiple stop–carry on cassettes (Luke *et al.*, 2008).

## Alternatives to non-canonical translation

Although this review has focused on the unusual translational mechanisms that viruses employ to cope with the unique constraints imposed by their compact genomes and atypical mRNAs, a number of RNA viruses have evolved various non-translational mechanisms that in some ways achieve similar results. As discussed previously, many viruses make use of sgRNAs, segmented genomes and post-translationally cleaved polyproteins in order to express the multiple proteins necessary for their replicative cycle. In addition, retro-transcribing viruses, besides a small proportion of RNA viruses, including orthomyxoviruses (e.g. influenzavirus) and bornaviruses, enter the host-cell nucleus where they make use of the host-cell splicing machinery for producing alternative transcripts.

An interesting parallel may be drawn between ribosomal frameshifting and a completely different mechanism – transcriptional slippage – that is utilized by several monopartite negative-strand RNA viruses (order *Mononegavirales*), notably viruses in the subfamily *Paramyxovirinae*, which includes measles virus (genus *Morbillivirus*), mumps virus (genus *Rubulavirus*) and parainfluenzaviruses (genus *Respirovirus*). In these viruses, programmed polymerase slippage or stuttering occurs at a specific site (3′-U*_n_*C*_m_*-5′, transcribed to A*_n_*G*_m_* in the mRNA; *n*+*m*≥8) during transcription of the phosphoprotein mRNAs, leading to the efficient insertion of one or more extra Gs in a proportion of transcripts (reviewed by Kolakofsky *et al.*, 2005). Thus, translation of the mRNAs leads to the production of different N-terminally coincident, C-terminally distinct proteins, with the relative proportion determined by species-specific details of the stuttering site. Transcriptional slippage is also used in the glycoprotein gene of ebolaviruses (family *Filoviridae*, order *Mononegavirales*; Sanchez *et al.*, 1996; Volchkova *et al.*, 2011).

A similar parallel may be made between translational codon redefinition (including stop-codon readthrough) and programmed RNA editing. The most common form of RNA editing in vertebrates is editing of adenosine to inosine by ADARs (adenosine deaminases acting on RNA; reviewed by Wulff & Nishikura, 2010). Inosine is read by the translational apparatus as guanosine; thus, for example, a templated UAG stop codon may subsequently be edited to UIG in a proportion of mRNA transcripts and translated as tryptophan. A slight variation of this mechanism is utilized by hepatitis delta virus – a subviral RNA satellite that replicates nuclearly and is dependent on hepatitis B virus for envelope proteins and, unusually, on cellular (normally DNA-dependent) RNA polymerase II for replication and transcription. The hepatitis delta virus genome is only known to encode one protein, known as δAg (delta antigen), which is translated in two forms of 24 and 27 kDa, both of which are essential (reviewed by Taylor, 2006). The larger form, which is required for virion assembly, has a 19–20 aa C-terminal extension and is produced late in infection upon ADAR editing of the coding-sense antigenome. The edit is copied to new genome-sense RNA and leads to the replacement of a UAG stop codon with a UGG tryptophan codon in transcribed mRNAs (Polson *et al.*, 1996).

## Concluding remarks

Together, it is clear that RNA viruses provide a fascinating plethora of examples of non-standard mechanisms for gene expression. Traditionally, virus research has focused on those species that are most relevant to humankind – that is, human viruses and the viruses of commercially important plants and animals, besides viruses of human parasites such as *Leishmania* and *Trichomonas*, and viruses of a few model organisms such as yeast and *Drosophila*. Recent years, however, have seen an explosion in the rate of acquisition of new sequencing data and, as sequencing turns more to environmental samples, there is the opportunity to sample viruses from a much larger diversity of hosts (e.g. diverse fungi, insects and protists). Some such viruses are highly divergent from known viruses and are likely to provide examples of new translational mechanisms, besides many new variations of previously identified mechanisms. This is particularly so for viruses of organisms (or organelles) with unusual components of the translational apparatus, such as unusual ribosomes or unusual tRNA types and abundances. Other newly discovered viruses are related more closely to known viruses but can provide a broader phylogenetic baseline for computational comparative analyses that can be used to detect undiscovered elements in economically and medically important species.

The discovery of novel translational elements is increasingly driven by bioinformatic analysis of sequence databases, but new data resources (e.g. whole-proteome mass spectrometry and whole-transcriptome ribosome profiling; Ingolia *et al.*, 2011) will play an increasingly important role. Although non-canonical translation appears to be less significant in DNA viruses and cellular genes, there are still many such examples ranging from the rather common presence of short ORFs in 5′ leaders to the highly conserved and functionally critical frameshift sites in release factor 2 and antizyme genes. The identification and characterization of novel types of non-canonical translation in RNA viruses will aid in cellular genome annotation by building a catalogue of biologically feasible mechanisms, and generating ‘search patterns’ that can be used as part of automated annotation pipelines for both cellular and viral genomes. In this review, we have included some examples where experimental details remain uncertain (e.g. hypothesized −2 frameshifting in a trichomonasvirus and +2 frameshifting in potyviruses) because we believe that it is useful for annotators of viral genomes to be aware of such possibilities.

Exceptions to the canonical translational rules can be programmed or incidental. The latter may be thought of as translational noise and occur in probably all genes for a small proportion of translating ribosomes. However, they are not subject to strong purifying selection so are generally not phylogenetically preserved over significant evolutionary distances. On the other hand, programmed exceptions generally (though not always) involve a significant proportion of translating ribosomes and tend to be subject to strong purifying selection and phylogenetic conservation. Not surprisingly, there are continua in the dimensions of efficiency, functionality and evolutionary conservation, and it is not always obvious whether a given case of non-canonical translation is programmed or incidental.

The study of non-canonical translation can lead to the development of extremely valuable tools for molecular biological research and biotechnology. A case in point is the use of stop–carry on cassettes for equimolar co-expression of multiple proteins from a single transcript. Non-canonical translational mechanisms (in particular frameshifting in HIV) have also been proposed as potential targets for antiviral drugs. This is particularly attractive if it can be demonstrated that a given mechanism is not utilized for host gene expression. Finally, by acquiring a greater understanding of the extent to which the translational machinery can be subverted from canonical cap-dependent scanning initiation and triplet decoding, and the mechanisms for achieving such subversions, one will also develop a greater understanding of the canonical mechanisms of eukaryotic translation – arguably (together with counterparts in bacteria and archaea) the most important process in the modern biosphere.
